# Dr. Arnold on Diseases of Troops

**Published:** 1811-01

**Authors:** Joseph Arnold

**Affiliations:** Suffolk


### *To the Editors of the Medical and Physical Journal.*

GENTLEMEN,

I WILLINGLY present to you the following simple narrative of facts, deeming them so unusual and eventually fortunate, considering the adverse circumstances under which they happened, as to deserve a more general publication than could be made, otherwise than by publishing them in your excellent Journal. Medical books are full of instances of extraordinary mortality prevailing in camps, prisons, and ships, whenever a contagious disease has broken out; these dreadful instances of visitation have often occurred in the West-Indies in our own times, and more recently were known to have taken place in the fleet of Sweden in the years 1808-9, as well as in the English ships that brought the army home from Spain after the battle of Corunna in 1809.

In the ensuing relation a disease manifestly and doubtlessly contagious, prevailed in a fifty-gun ship (armed en flute) in which, besides the ship's company, were embarked 343 soldiers, and upwards of 130 women and children.—Among this large and crowded collection of human beings, dysentery appeared, and gradually spread to an alarming extent, so that at different times, 178 persons came under the care of the surgeon for this complaint alone; and of this vast number ber (I speak it with admiration), only two men could be said to die of the disorder itself, and two others of its effects, by which marasmus and death were at length induced.* If, therefore it should accord with your wishes to make known

| Died on board. | Numbers. | Diseases. |
|---|---|---|
| | 78 | Febres. |
| | 22 | Cynanche. |
| | 26 | Pneumonia. |
| | 43 | Ophthalmia. |
| 1 | 5 | Phthisis. |
| | 1 | Enterites |
| | 3 | Hepatitis. |
| | 2 | Nephritis. |
| | 10 | Rheumatismus. |
| | 1 | Podagra. |
| | 13 | Erythema. |
| | 2 | Hemoptysis. |
| | 3 | Hæmorrhois. |
| | 10 | Catarrhus. |
| 2 | 178 | Dysenteria |
| | 1 | Syncope. |
| | 7 | Dyspepsia. |
| | 2 | Epilepsia. |
| 1 | 2 | Asthma. |
| | 26 | Colica. |
| | 17 | Cholera. |
| | 24 | Diarrhæa. |
| | 5 | Hydrops. |
| | 3 | Scrofula. |
| | 14 | Syphilis. |
| | 14 | Scorbutus. |
| | 17 | Icterus. |
| | 4 | Hernia. |
| 2 | 2 | Tabes mesenterica. |
| | 106 | Morbi irregulares. |
| 1 | 46 | Vulnera. |
| | 16 | Ulcera. |

* *The following is the Number of Sick, both Soldiers and Sailors, who came under the Treatment of the Naval Surgeon, between May the 22d, and December the 28th.*

the causes of this success, and the means employed to ensure it, an insertion of the following events which came to my knowledge will answer that purpose.

"His Majesty's ——— regiment embarked on board a ship of war (a two-decker) about the 9th of May 809, to be conveyed to a very distant part of the world: they were composed for the most part of fine young men recruited from different parts of the kingdom, and remained on board the ship about a fortnight before the final orders came for sailing; during which time some of the soldiers, who had been in an hospital, were so far recovered from fever and dysentery, as to be thought capable of proceeding on their voyage; others, however, were found so ill as to require to be relanded, which however were few in number, and were taken to a military hospital by the medical attendant attached to that wing of the regiment which was embarked in the ship.

On the 22d of May the ship, with a consort containing the other part of the battalion, sailed from St. Helen's, and proceeded down Channel with a fair wind.

It may be necessary to remark, that when troops are embarked on board ships of war, the military and naval surgeons perform their respective duties independently of each other, unless that the naval surgeon more particularly reports to the Captain of the ship such circumstances in the economy of the vessel as may have influence on the *general* health of the people on board; but the individual treatment of the patients depends entirely on the respective surgeons of the two departments. Hence, although it is of delicate import for the naval surgeon to interfere with the soldiers without his advice being required by the military attendant, yet it behoves him especially to be attentive to the general state of the sick of *both* departments. For which reason, a complaint occurring, certainly, or even doubtfully contagious, being so alarming and of such great importance to every person in the ship, comes so far under the control of the naval surgeon as to render it necessary for him to interfere, in order as soon as possible to put a stop to the complaint, or to prevent its communication with the sailors.

The weather was moderate for the first week, but about the 30th of May a heavy gale was encountered, by which the ship was completely drenched with water, and the soldiers much exposed to wet and other severe suffering in the Bay of Biscay. The storm remitted on the following day, but another equal in violence took place three days after; and again on the 4th of June another tremendous storm added to their sufferings, during which day, Cape Finisterre being on the lee, they were chased by a strange ship of a large size, as much sail was set as possible, and the lower deck ports being ill closed, a great quantity of water was admitted, so that the carpenter was obliged to scuttle the lower deck to let into the hold a vast wave of water dashing from side to side of the ship among nearly four hundred men, women, and children. A remission was experienced on the fifth, and on the 12th the ship anchored in Funchal roads, many of the men being sick, and having in this short but severe passage become affected with the scorbutic diathesis.

When at anchor in this place the surgeon of the ship was surprised with the complaints of one of the soldier's wives, whom he found actually labouring under a severe attack of dysentery, with which she had been affected several days; and in a few days more two other women made complaint of the same symptoms. And to those who are acquainted with naval practice, it will not appear strange, that the disorder first appearing should continue to spread among the *women*, as they are all obliged to resort to the same necessary convenience, by means of which the complaint is more generally propagated.

The Surgeon of the ship deeming it of the greatest consequence *strenuously* to endeavour to put a stop to the further progress of the disease, by separation of the sick, advised that the women affected should be sent on shore at Madeira; but the individual hardship of separating the wives from their husbands, perhaps under such circumstances precluding them from ever meeting again, so far prevailed upon the humanity of the Commander, that they were ordered to remain on board, and the only then preventative consisted in keeping the women as little communicating with other persons as possible; but how ineffectual an entire separation was, must appear, when it is stated that there was not room for the inhabitants of the lower deck even to hang up their hammocks without absolute contact, many being compelled to place their bedding upon the wet deck.

The ship sailed from Madeira (the people having been refreshed with fruit, vegetables, and fresh beef) on the 19th of June, and on the 29th arrived at Porto Praya, on the island of St. Jago, from which they procured a stock of live cattle, and then proceeded on their voyage southward. On the 22d of June the armourer of the ship made complaint of severe dysenteric symptoms, and it is remarkable that this was almost the only sailor who had his birth on the lower deck among the soldiers, and he was placed very near the woman first affected with the disorder, for it is really impossible to prevent offensive matter being left about in a ship in boisterous weather, when all is darkness between decks, and scarcely scarcely a healthy person is able to proceed from one part of the ship to the other, unless upon his hands and knees; and much less a poor sick woman from her hammock on the lower deck to the round-house, on the frequent occasions that take place in this complaint.

As might be expected, the malady having once appeared gradually increased both among soldiers and sailors; by the 6th of August, being then in South latitude 23°, West longitude 38°, twenty-six of the ship's company had been in various degrees attacked by the complaint, most of whose symptoms could be altogether removed or relieved by the remedies employed by the naval surgeon; there being only two of the sailors so ill as to take to their hammocks, and eight others under less severe symptoms.

At this time the most that the surgeon of the ship could do was, to permit little communication between the decks; the military having exclusively the lower, the sailors the gun-deck. The reports of the sick daily made by the respective medical attendants to their commanding officers being compared, there was found to be a great disparity, both in the numbers affected, and in the severity of their complaints; for while of the sailors there were but ten sick of dysentery, (the others previously having recovered from the disorder) there were forty-five of the soldiers; and of the former two only were confined to their beds, but there were twenty-seven of the latter: it was therefore determined by the respective commanding officers, to place the sick, both soldiers and sailors, under the care of the naval surgeon alone. This being enforced by a written order from the captain of the ship, aided by a regimental order from the colonel, entire authority was placed in his hands as far as related to the medical treatment of the persons then sick, or should at any future part of the voyage become so.

In obedience to these orders, the surgeon of the ship (being really placed in uncommon circumstances, having a very serious and arduous task to perform, and being endowed with double responsibility) immediately commenced his duty among the soldiers who he had never before individually visited, and with whose previous situation he was but little acquainted, unless from the written reports of their medical attendant, which he had sometimes an opportunity of seeing.

He found twenty-seven persons in their hammocks, men, women, and children, labouring under different stages of dysentery; some with the primary symptoms; others with the consequences of the disorder, emaciation, extreme debility, lity, and severe abdominal pain. Besides those that were confined to bed, there were about as many others, less seriously, or more recently attacked, but labouring under the same disease.

Things being so situated, he prescribed such remedies as seemed to him necessary, and advised, that as the ship was within two days sail of Rio de Janeiro, she should proceed thither, where could be had every kind of refreshment, and where was an English fleet and a naval hospital.

As soon as the ship entered this harbour an admiral's order was procured to remove the more seriously sick to the naval hospital, and that every person, even the most slightly affected with the complaint, should be put on board a transport lying in the harbour, by which means the naval surgeon hoped to be enabled to give so effectual a check to the complaint as to be soon enabled to put a stop to it entirely.

When in Rio de Janeiro, provisions of every sort were sent to the ships, and after the sick were removed, a few slight cases of the disorder only appeared, which were no sooner seen than they were sent out of the ship, and in ten days no further case of the disease intervened.

On the 24th of August, having remained three weeks in this port, it was determined to proceed again to sea, having lost one old man only (who appeared to be moribund when sent there) at the hospital. The sick were then all returned on board from the transport in a state of convalescence; but those from the hospital very severely affected, for the most part with the consequences of the disease, rather than with the real complaint: and eight or ten men, with two women far advanced in pregnancy, were too ill to remain out of their hammocks. It was hoped, that, by departing somewhat south of the tropic of Capricorn, the heat hitherto experienced in the torrid zone, and which was thought to favour the complaint, would be obviated, and thus no further recurrence would take place; and this in a remarkable degree *was* observed, not more than ten or twelve cases appearing after that time; and those that returned from the hospital of St. Sebastian continued to recover.

The weather, during the passage from the Brazils to the Cape of Good Hope, being comparatively cold, (between the tropics it had been generally between 90 and 98 degrees of Fahrenheit, whereas here it was often under 60) few diseases, and these apparently arising solely from cold, were observed; some of these much resembled the former complaint. Many of the men also (having on their departure from England not been furnished with any warm cloathing) were attacked tacked with very severe pneumonic symptoms; no *fatal* cases, however, occurred, although frequent phlebotomy was in several found necessary.

On the 22d of September the ship anchored in Table Bay, in a very healthy state, there being no remains of the former alarming disorder.

After being refreshed at the Cape, the ship departed on the 15th of October, but about the 5th of November (south lat. 36 deg. 16 min. east long. 53 deg. 40 min.) the surgeon was extremely anxious, on observing that several men, to the number of eight or ten, made complaint of disordered bowels, accompanied with griping tenesmus, and frequent scanty mucous alvine evacuations. These complaints continued to be made for several succeeding days, but very easily giving way to medicine, and being for the most part unaccompanied with pyrexia, he was inclined to believe they proceeded from some other cause than contagion. The men, on leaving the Cape, had had a change in their diet; having formerly had rum, but now wine, which is made at the Cape, and which is generally drank too new; and it is a common observation there, that it is often productive of these disorders; he therefore advised that half allowance of grog should be served out, and this seemed to have the intended good effect; but few further instances of sickness appearing during the time the ship continued on her voyage, which terminated on the 28th of December.

Thus after a voyage of vast extent, approaching to near 20,000 miles, in most of the climates of the globe, in a crowded ship, during which time upwards of 600 persons applied to the surgeon for assistance, many of them with a disease manifestly contagious, often dangerous, and whose presence has often in camps and ships been synonymous with death and desolation; so little was the mortality that only two persons could be said absolutely to die of the disease itself, and two others of its consequences, mesenteric obstruction and marasmus.

Having now therefore described the events as they occurred, and stated that only four persons died, whose deaths proceeded from the disease in question, I come to mention by what means this success was produced; what were the attempts of the surgeon to stop its progress; and what his treatment of the disease when it actually appeared.

When a disease of an infectious nature prevails in *any* situation and spreads much, there is great reason to suppose that there either is some neglect or mismanagement, or that the necessary preventatives are out of the reach of the medical attendant, which latter circumstance is much more frequent on board ships than on shore, seeing that a separation of the sick from the healthy is there but slightly practicable. If, however, an actual non-intercourse cannot be effected, it ought, as much as possible, to be approached to; hence, when the surgeon of the ship was ordered to attend the military, and found nearly thirty men confined to their beds with the disease, in different parts of the ship, he ordered them to be conveyed forward, and permitted no person but the medical attendants and servants to have intercourse with them; and aided by the commanding officers, a line of demarkation was strictly kept on both sides the deck by sentries. When the weather would permit the ports were kept open, and in the night windsails were well attended by sentries at the different hatchways, who were kept to their duty by the frequent visitations of the military officer having the watch; the deck under the hammocks was frequently sprinkled with vinegar, and a fumigating lamp suspended over the fore peak scuttle, and kept almost constantly burning.

And here it may be necessary to mention, that the naval surgeon discovered a source of evil, that evidently prevailed much in favouring the spreading of the disease among the persons in the lower deck. Many of the sick being too ill to reach the necessary on the upper deck, and having the frequent calls for evacuation so common in this complaint, had been furnished with small tubs for this purpose; and these most unwisely were unprovided with covers, from whence arose a continual source of danger; these conveniences, therefore, were immediately provided with such covers as could not be left open, and men were ordered very frequently to wash out these utensils with sea-water. Alterations also were made in the head of the ship, to which the healthy men resorted, to prevent any lodgement of stercoraceous matter; and one side of it was kept for the healthy, and the other for such of the sick as were able to come upon deck. Small conveniences for the same purpose were made in the fore-chains, so contrived, as easily to be removed as soon as no longer required; and to these salutary alterations more than to any thing else, the surgeon of the ship attributed the fortunate change, that took place soon after the ship left the Brazil coast.

With respect to the medical treatment little need be said, there being nothing unusual in it, nor did the surgeon follow any *particular* practice, or prescribe any thing more than is furnished to every ship of war; calomel, opium, neutral salts, magnesia, ipecacuanha, castor-oil, jalap, senna, and quassia amara, form nearly all the materia medica employed for for the cure of this disease, and he has reason to believe, that a judicious exhibition of these alone will in most cases effect a cure, though he has seen, from a misapplication of them, the worst and most dangerous effects. All these medicines were employed according to circumstances, but it would be impossible to give such an account of their effects, as to form rules for future practice, this disease being so variable in its progress, and so often requiring sudden changes of treatment. Nor would he enter into a disquisition on the pathology of the complaint, or mention the different opinions held by medical men on the nature of the disease, or the different modes of treatment that have been proposed. No one can doubt from the symptoms, the causes, and the appearances on dissection, that it consisted in a specific inflammation of some part or other of the intestines, by which their functions are injured or destroyed, and the progressive impulse of their contents impeded; and being affected with a morbidly increased sensibility from their inner coats being abraded, the patients often, particularly if the included matter be hard or scybalous, are thrown into extreme torture. This complaint, he believes, like most, if not all of the exanthemata that depend upon specific inflammation, has its commencement, increase and decline at certain periods, at the end of which the specific action ceases: and though the painful symptoms often continue for a great length of time, nearly as acute as during the prevalence of the complaint itself, yet they are occasioned by the abrasion of the intestines, which are sometimes left inflamed, ulcerated, or scirrhous, and these protracted symptoms are often very difficultly removed and sometimes prove fatal, being attended with almost continual pain, emaciation, debility and hectic, which at length terminate the sufferings of the patient, unless he be previously cut off by an active and more general inflammation induced by the obstructions of the mesentery.

To mention the medicines above spoken of, *individually*, it may be stated, that *calomel* was often used as a purge, with jalap, senna, salts, or ipecacuanha, sometimes as an alterative with opium, or mercurial pill. In the more ordinary cases, a few doses of laxative medicines would effect a cure; in others the disease was more complicated. In a very few cases, the surgeon was obliged to resort to ptyalism, which had the desired effect; but he repaired to this as seldom as possible, and only in the most severe instances, as the patients were by it much reduced, and the recuperative means in his power very scanty. In some cases it was found adviseable to induce a copious sweat, and this was generally done by Dover's

Dover's powder and calomel; when the perspiration was sufficiently profuse, it was very beneficial.

*Opium* was often found necessary, and however it may have been reprobated by some physicians, seemed not a little to have been required, to relieve the inordinate action and long continued distress, under which some of the patients suffered; he had occasion, however, in a few instances, to observe its deleterious effects when improperly prescribed. *Neutral salts* were a common purgative employed at the beginning of the disease, sometimes with calomel and senna; and a few drops of sulphuric acid, or two ounces of lime juice, were found an excellent addition.

*Ipecacuanha* has been a common remedy in this disease; by some reprobated, by others extolled, but ought not to be withheld. It was found beneficial when combined with opium as a sudorific, and with rhubarb as a purge, and the most remarkable and unequivocal good effect, arose from a bihorial repetition of half a grain of ipecacuanha with two of rhubarb, the belly having been previously fully evacuated by more active cathartics.

From *magnesia* was often observed the greatest relief; it sometimes occurred, that patients in the protracted stages of the disorder suffered with severe gastrodynia, and spasmodic affections of the abdominal viscera, which were often relieved by this medicine and oleum menthæ piperitæ alone, sometimes with opium. In two cases the most excruciating agony, accompanied with violent convulsions terrible to be looked upon, the patients mean while howling with intolerable suffering, were relieved and soon removed by these medicines.

*Jalap* was sometimes employed with calomel in the more trivial cases, or on the first onset, and had the best effects, but it was evidently too rough when the complaint was fully formed.

Of all the purgatives, *castor oil* was found in the advanced stage of the disorder to be the safest, most effectual, and the easiest of operation; there was scarcely a doubt, but that the life of one man was actually preserved by repeated doses of it, every other purgative throwing him into the most dreadful convulsions.

These were the medicines principally employed during the disease itself; when its symptoms went off, and the patient was left weak, emaciated, and often at death's door, the intention was to strengthen the stomach, and by sympathy with it the other functions of the body; and this was very effectually done by a weak infusion of quassia amara, to which was added a small quantity of zingiber. By these means means nearly all the patients who suffered with dysentery recovered, and its further progress was prevented."

Thus, Gentlemen, I have related, as they are reported to have occurred, a faithful history of the events that happened lately on board one of his Majesty's ships, and as they were of very unusual and fortunate event, seeing that during a very long voyage, performed in eight months, only seven persons died, one by an accident, and another of asthma, who was an old invalid; I would from a revision of the circumstances inculcate, that however adverse may be the circumstances, whatever opposition may be made to his attempts, or whatever obstructions should oppose the efforts of the medical man, when called upon to give his assistance in such cases, still he ought to embrace every, even the slightest means of ensuring success, and thus in many cases of almost hopeless good event, prosperity will ultimately crown his efforts.

I have the honour to be,

With the greatest respect,

*Suffolk, Nov.* 10, 1810. Your obedient servant,

JOSEPH ARNOLD, M. D. &c.

